# Enhancing the protection of influenza virus vaccines with BECC TLR4 adjuvant in aged mice

**DOI:** 10.1038/s41598-023-27965-x

**Published:** 2023-01-13

**Authors:** Robert Haupt, Lauren Baracco, Erin M. Harberts, Madhumathi Loganathan, Lucas J. Kerstetter, Florian Krammer, Lynda Coughlan, Robert K. Ernst, Matthew B. Frieman

**Affiliations:** 1 Department of Microbiology and Immunology, School of Medicine, University of Maryland, Baltimore, MD USA; 2 Center for Pathogen Research, School of Medicine, University of Maryland, Baltimore, MD USA; 3 Department of Microbial Pathogenesis, School of Dentistry, University of Maryland, Baltimore, MD USA; 4 Department of Microbiology, Icahn School of Medicine, New York, NY USA; 5 Center for Vaccine Research and Pandemic Preparedness (C-VARPP), Icahn School of Medicine at Mount Sinai, New York, NY USA; 6 Department of Pathology, Molecular and Cell Based Medicine, Icahn School of Medicine at Mount Sinai, New York, NY USA; 7 Center for Vaccine Development and Global Health (CVD), University of Maryland School of Medicine, Baltimore, MD 21201 USA; 8 Present Address: Therapeutic Discovery Branch, Molecular Biology Division, USAMRIID, Fort Detrick, MD USA

**Keywords:** Vaccines, Viral infection

## Abstract

Influenza A virus (IAV) is a leading cause of respiratory disease worldwide often resulting in severe morbidity and mortality. We have previously shown that the Bacterial Enzymatic Combinatorial Chemistry (BECC) adjuvants, BECC438 and BECC470, formulated with an influenza virus hemagglutinin (HA) protein vaccine, offer greater protection from influenza virus challenge in mouse respiratory models using adult mice than standard HA:adjuvant combinations. In this study, we determined that immunization with HA + BECC adjuvants also significantly broadened the epitopes targeted on HA as compared with other adjuvants, resulting in increased titers of antibodies directed against the highly conserved HA stalk domain. Importantly, we demonstrate that BECC470 combined with an influenza virus HA protein antigen in a prime-only immunization regimen was able to achieve complete protection from challenge in a ~ 12-month-old mouse aged model. Together, this demonstrates the heightened protection provided by the BECC470 adjuvant in an influenza virus vaccine model and shows the enhanced immune response, as compared to other adjuvants elicited by the formulation of HA with BECC470.

## Introduction

Influenza viruses cause mild to severe respiratory infections in humans and are a major public health problem. According to the World Health Organization, seasonal influenza viruses, including the H1N1 and H3N2 influenza A viruses (IAV), as well as influenza B viruses, cause approximately 3–5 million severe cases and 290,000–650,000 deaths each year worldwide^1^. The standard quadrivalent influenza virus vaccine most prevalent in use today in the US consists of four different HA antigens (15 μg per HA antigen, depending on vaccine formulation) derived from individual influenza viruses; two influenza A viruses and two influenza B viruses and is administered to individuals greater than 6 months of age and is normally unadjuvanted^2^. The Center for Disease Control (CDC) reports that vaccination reduces the risk of influenza illness by between 40 and 60% among the overall population during seasons when most circulating influenza viruses are well-matched to the influenza vaccine^3^.

Disadvantages of current seasonal influenza vaccines are the fact that they elicit largely strain-specific antibody responses directed towards the antigenically variable HA head domain. In addition to the globular head domain, the HA trimer possesses a highly conserved stalk domain. The amino acid sequence of the HA stalk is reasonably well conserved between distinct influenza viruses, and it is therefore a major target for universal influenza virus vaccines^4^. As such, there is great interest in exploring adjuvants which could increase both the magnitude and breadth of the humoral immune response elicited by conventional HA-based vaccines. Older adults display significantly reduced influenza-specific antibody responses compared with young adults and/or fail to maintain durable antibody titers indicative of immune protection (termed “seroprotection”)^5–10^. For individuals 65 and older, Fluzone high-dose quadrivalent includes a dose of 60 μg of HA, or FLUAD™, specifically designed as a trivalent vaccine (now also available as a quadrivalent) with a standard dose of the HA antigen (15 μg of each HA antigen) formulated with the adjuvant MF59^11,12^. MF59 is an oil-in-water emulsion of squalene oil, which helps create a more potent and durable immune response after vaccination in elderly individuals^13^.

### Adjuvants boost immune protection

Adjuvants are components used in vaccines to enhance an immune response^14^. Recombinant protein-based vaccines in general as well as some inactivated viral vaccines—especially when split or partially purified, while more tolerable to the vaccinee, are often poorly immunogenic and require additional components to help stimulate the production of protective antibodies and effector T cell functions^15^. These vaccines can be formulated with adjuvants to enhance their immunogenicity.

We have used the Bacterial Enzymatic Combinatorial Chemistry (BECC) technology to generate novel lipid A based adjuvants^16,17^. More specifically, BECC438 and BECC470 were derived from the backbone structure of non-immunogenic *Y. pestis* lipid A and screened using reporter cell lines and flow cytometry for the ability to activate NFκB and cytokine production^13,14^. BECC438 is bis-phosphorylated (1 and 4′ position) with two secondary C16 acyl-chains at the 2 and 2′ positions^14,15^. BECC470 is mono-phosphorylated (1′ position) and has a C14 secondary acyl-chain added at the 4′ position along with a secondary C16 acyl-chain at the 2 position. These differences in BECC adjuvant lipid A structure have been shown to stimulate an innate immune response greater than phosphorylated hexa-acyl disaccharide (PHAD), a monophosphorylated lipid A, but less than *E. coli*^16,17^.

Previously, we showed that novel BECC-derived BECC438 and BECC470 stimulate a balanced Th1/Th2 immune response and elicit protection from homologous influenza virus infection and in 6–8-week-old mice, with either prime-boost or prime only vaccination schedule^18^. BECC’s balanced response provided superior protection from weight loss, lung viral titer reduction, and reduction of adverse lung pathology, when compared to the Th2-driven adjuvant alhydrogel (alum), an aluminum salt or Th1-driven PHAD, a toll like receptor (TLR) 4 ligand and synthetic monophosphoryl lipid A (4′ position).

In this manuscript, we demonstrate that BECC470 combined with an influenza virus HA from A/California/04/09 (Cal/09, H1N1) is able to protect ~ 12-month-old mice using a prime only or prime/boost vaccination schedule, that passive transfer of antibody from vaccinated mice can partially protect naïve mice, and that the BECC470 adjuvant broadens antibody responses towards conserved epitopes on the HA stalk. Identifying vaccine candidates or formulations which can elicit antibodies directed towards the HA stalk domain, is a major goal for universal influenza virus vaccine development^19,20^. We find that a significant increase in stalk binding antibodies are induced with BECC470/HA vaccination compared to other adjuvants combined with the same HA antigen. Together this demonstrates the unique effect of the BECC470 adjuvant in an influenza virus vaccine model in mice.

## Results

### Protection in aged mice with BECC-adjuvanted vaccine

Using young adult (6–8-week-old) mice, we previously reported protection from IAV challenge after vaccination with H1 (A/California/04/2009) formulated with BECC438 and BECC470^16^; 0.04 μg rHA elicited protection from homologous challenge in formulations adjuvanted with 50 μg BECC438 or BECC470, unlike those adjuvanted with 100 μg of alum or 50 μg PHAD^16^. As a follow-up to these experiments, we wanted to assess adjuvant induced protection in aged (~ 12-month-old) mice to determine if immunosenescence observed in aging could be recovered through the use of the BECC-based adjuvant system.

**Figure 1:**
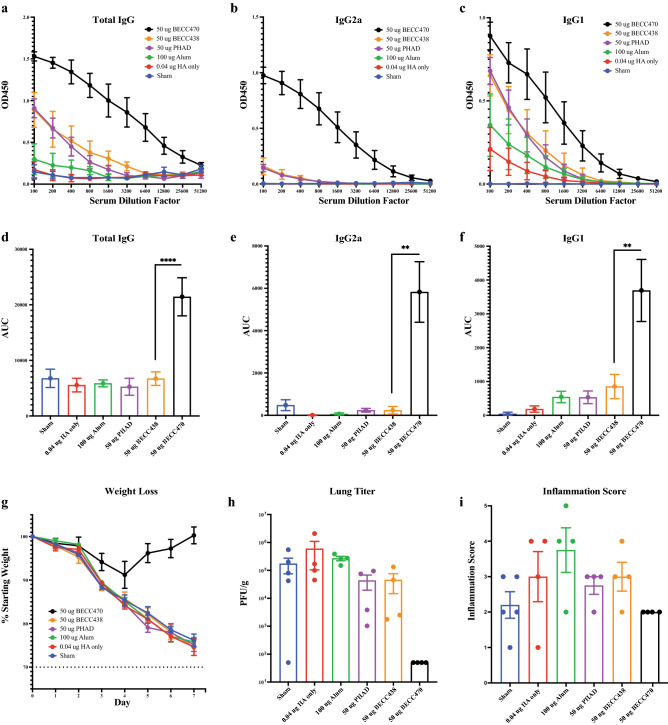
Protection of aged mice following prime + boost vaccination (0.04 μg HA). (**a**) Pre-infection day 28 H1-specific serum ELISA total IgG, (**b**) isotype subclass specific IgG2a and (**c**) IgG1 of aged BALB/c mice vaccinated with 0.04 µg Cal/09 rHA protein in combination with 100 µg alum, 50 µg PHAD, 50 BECC438 or 50 µg BECC470 adjuvant in prime + boost schedule (mean ± SEM). Area under the curve (AUC) (mean ± standard error of the mean) of above serum ELISA curves for (**d**) total IgG (****p < 0.0001), (**e**) IgG2a (**p = 0.0022) and (**f**) IgG1 (**p = 0.0013). (**g**) 7-day weight loss in BALB/c mice (5 per group) after infection with 32,000 PFU of NL/09 (mean ± SEM). (**h**) Virus titer of lung homogenate 7-days post infection (mean ± SEM) with (**i**) pathology inflammation scoring of lung histology slides (mean ± SEM). Prism 9 used to calculate mean + SEM and AUC ± standard error.

To assess protection in aged animals, we prime-boost vaccinated groups of mice (5/group) with 0.04 μg rHA in combination with 100 μg alum, 50 μg PHAD, 50 μg BECC438, or 50 μg BECC470 and measured H1-specific antibody responses by enzyme-linked immunosorbent assay (ELISA). While total and isotype subclass specific H1-specific IgG titers from day 28 sera showed high titers of BECC-induced antibody in the younger adult mice (not shown), in aged mice only the BECC470 adjuvanted vaccine stimulated a strong response in H1-specific total IgG (p < 0.0001), IgG2a (p = 0.0022) and IgG1 (p = 0.0013) (Fig. 1a–c enzyme-linked immunosorbent assay (ELISA) line graphs and corresponding Fig. 1d–f AUC bar graphs). After homologous challenge with 32,000 plaque forming units (PFU) of IAV A/Netherlands/602/09 (NL/09, H1N1, antigenically similar to Cal/09, all groups of aged mice including the BECC438 group, lost weight similar to the sham infected cohort with the exception of those vaccinated with the BECC470 adjuvant (Fig. 1g). Correspondingly, significant lung viral titers were detected in all groups except those mice vaccinated with BECC470 (Fig. 1h). Despite reduced lung viral titers, histology and inflammation scoring showed some moderate bronchiolar and periarterial inflammation in the BECC470 group (Supplemental Fig. 1 and Fig. 1i).

### Prime only dose of BECC-adjuvanted vaccine provides protection in aged mice

**Figure 2:**
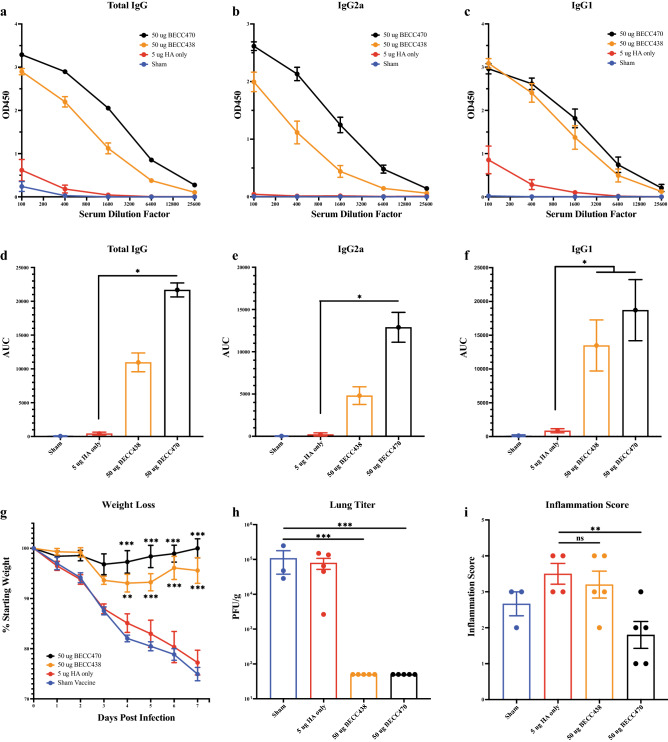
Protection of aged mice following prime-only vaccination (5 μg HA). (**a**) Pre-infection day 28 H1-specific serum ELISA total IgG, (**b**) isotype subclass specific IgG2a and (**c**) IgG1 of aged BALB/c mice vaccinated with 5 µg Cal/09 rHA protein in combination with 50 BECC438 or 50 µg BECC470 adjuvant in prime only schedule (mean ± SEM). (**b**) Area under the curve (AUC) (mean ± standard error) of ELISA curves for (**d**) total IgG (*p = 0.0258), (**e**) IgG2a (*p = 0.0484) and (**f**) IgG1 (*p = 0.0259). (**g**) 7-day weight loss in BALB/c mice (5 per group) after infection with 32,000 PFU of NL/09 (mean ± SEM). (**h**) Virus titer of lung homogenate 7-days post infection (mean ± SEM) with (**i**) pathology inflammation scoring of lung histology slides (mean ± SEM). Prism 9 used to calculate mean ± SEM and AUC ± standard error of the mean.

Previously, in 6–8-week-old mice, we showed that a single, prime-only dose of either BECC438 or BECC470-adjuvanted vaccine provides protection from homologous IAV challenge. In that study, mice displayed minimal to no weight loss when immunized with 5 μg rHA in combination with either 50 μg BECC438 or BECC470. In this study, we used a prime-only vaccination schedule to assess the immune response and/or protection with a single dose in an aged mouse cohort using 12-month-old, female, BALB/c, retired breeder mice (5/group). Mice were immunized with 5 μg of rHA in combination with either 50 μg BECC438 or BECC470. Serum from day 28 (pre-infection) vaccination groups was assessed for H1-specific total IgG (Fig. 2a,d) and isotype subclass specific IgG2a (Fig. 2b,e) and IgG1 (Fig. 2c,f). Total IgG, IgG2a and IgG1 antibody production was minimal in both the sham and rHA-only groups, however, significantly higher antibody titers were found in BECC470 adjuvanted groups (p = 0.0258, p = 0.0484 and p = 0.0259 respectively). At day 28, these aged mice were infected with 32,000 PFU of IAV H1N1 NL/09 and weighed for seven days. Protection from morbidity (i.e. weight loss) in BECC adjuvanted groups was superior to the sham and rHA-only groups, with minimal weight loss for + BECC groups as compared with > 20% weight loss for rHA-only and sham animals which were not protected. Interestingly, while prime-boost vaccination with 50 μg of BECC438 adjuvant in combination with 0.04 μg rHA was not protective in aged mice (as shown in Fig. 2), a single, day 0 vaccination with 50 μg of BECC438 adjuvant in combination with a higher dose of 5 μg rHA antigen was now sufficient to protect from weight loss. Again, BECC470 adjuvanted vaccine weight loss protection was superior to BECC438 (Fig. 2g). Virus titer was approximately 1 × 10^5^ PFU/g in both the sham and rHA only groups, whereas the BECC438 and BECC470 adjuvanted groups showed no detectable viral titer in day 7 lung homogenates (Fig. 2h). Histology for the sham group was similar to the rHA only group, with pronounced bronchiolar and periarterial inflammation, and moderate bronchiolar necrosis. These pathologies were similar in the BECC438 group with decreased infiltration in the alveolar spaces. All pathology, however, was mitigated in the BECC470 group when 5 μg rHA was used (Supplemental Fig. 2). Inflammation scores in all but the BECC470 adjuvanted group were likewise elevated according to pathology with reduced bronchiolar and periarterial inflammation observed for the BECC470 group (Fig. 2i).

### BECC adjuvant induced antibody reduces disease burden

**Figure 3:**
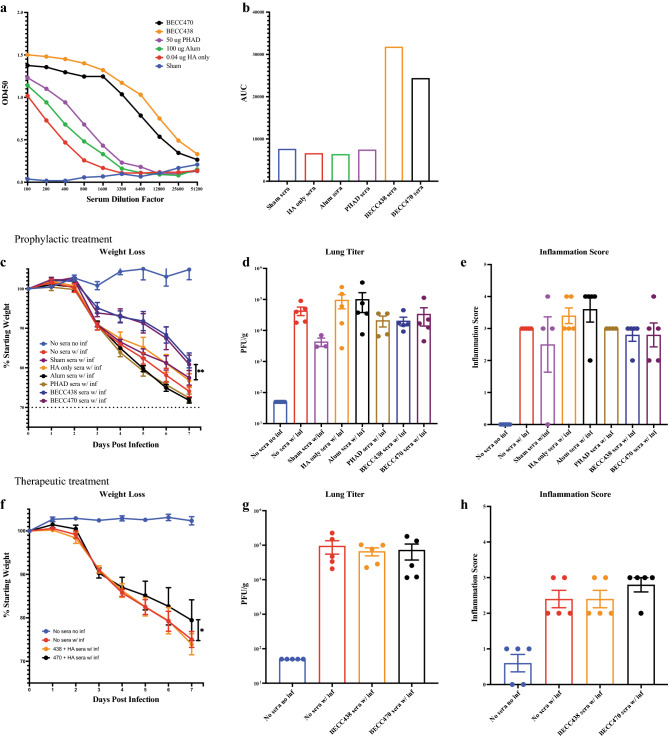
Protection from homologous H1N1 challenge with prophylactic or therapeutic passive transfer of vaccination sera. (**a**) Pooled, day-28 serum H1-specific ELISA total IgG of young adult BALB/c mice vaccinated with 0.04 µg Cal/09 rHA protein in combination with 100 µg alum, 50 µg PHAD 50 µg BECC438 or 50 µg BECC470 adjuvant in prime-boost schedule (mean ± SEM). (**b**) AUC of pooled, pre-infection total IgG antibody titer curve (mean). (**c**) 7-day weight loss (mean ± SEM) of BALB/c mice IP-injected with 100 µL vaccination sera (by group) and infected 2 h later with 3200 PFU NL/09 (**p = 0.008). (**d**) Virus titer (mean + SEM) of mouse lung homogenate in (**c**), 7-days post-infection. (**e**) Pathology inflammation scoring (mean ± SEM) of lung histology slides of mice in (**c**), 7-days post-infection. (**f**) 7-day weight loss (mean ± SEM) of BALB/c mice infected with 3200 PFU NL/09 and IP-injected with 100 µL vaccination sera (by group) at day 1 and day 3 post-infection (*p = 0.0288). (**g**) Virus titer (mean ± SEM) of mouse lung homogenate in (**f**), 7-days post-infection. (**h**) Pathology inflammation scoring (mean ± SEM) of mouse lung histology slides in (**f**), 7-days post-infection.

To test the protective capacity of antibody alone against weight loss, sera from vaccinated young adult mice was passively transferred intraperitoneally (IP) into groups of naïve, 6–8-week-old young adult BALB/c mice and these mice were then challenged with 3200 PFU of homologous IAV H1N1 NL/09. Serum from young adult mice was collected after a prime-boost immunization scheme (day 0 and day 14) with 0.04 μg rHA in combination with either 100 μg alum, 50 μg PHAD, 50 μg BECC438, or 50 μg BECC470. Briefly, whole blood was collected on day 28 via cardiac puncture, serum was separated and then pooled by group. Prior to transfer of pooled serum, we verified that H1-specific total IgG antibody titers. Total IgG levels were determined to be similar to sham in the rHA only, alum, and PHAD groups. The BECC adjuvanted groups, however, showed markedly higher H1-specific IgG antibody titers as compared to all other groups (Fig. 3a,b).

To test how vaccine sera could protect aged mice, we IP-injected 100 μL of untreated serum from the full range of vaccine groups into adult mice (5/group), waited for two hours for antibody absorption then i.n. infected the mice with 3200 PFU of IAV NL/09 virus. By day 7 post-infection, the mice that received sera from BECC438 and BECC470 vaccinated mice showed a significant difference (p = 0.008) in weight loss protection over the rHA-only vaccination group (Fig. 3c). Although there was greater weight loss with passive transfer of sera as compared to prime-boost vaccination (Fig. 2g), there was still a significant improvement in BECC-adjuvanted weight loss protection over the other infected groups, even with similar virus titer (Fig. 3d), lung infiltration (Supplemental Fig. 3), and inflammation (Fig. 3e) as compared to non-BECC groups. Passive transfer of 100 µL of sera shows at least partial protection from weight loss, suggesting that multiple parameters of the immune response may contribute to overall protection from challenge^21,22^.

We also evaluated the contribution of immune sera in a post-exposure, therapeutic model, in which we i.n. infected groups of adult mice (5/group) with 3200 PFU of IAV NL/09 virus and then IP-injected them with 100 μL of untreated BECC438 or BECC470 serum at day 1 and day 3 after infection (200 μL each mouse total). Mice given BECC470 convalescent serum showed significant protection from weight loss over the BECC438 group, which were similar to the sham “no sera” group (Fig. 3f). While the viral titers in all infected groups were similarly high (Fig. 3g), there was less (p = 0.0288) weight loss in the BECC470 group over those receiving BECC438 vaccine sera. Most striking are the open alveolar space inflammation in the BECC-470-adjuvanted H&E-stained slides, even with marked bronchiolar and periarterial inflammation. The No Sera and BECC438 groups showed marked inflammation and closed alveolar spaces (Fig. 3h and Supplemental Fig. 3).

### BECC adjuvant drives production of broadly protective HA stalk antibody in adult, but not aged mice

**Figure 4:**
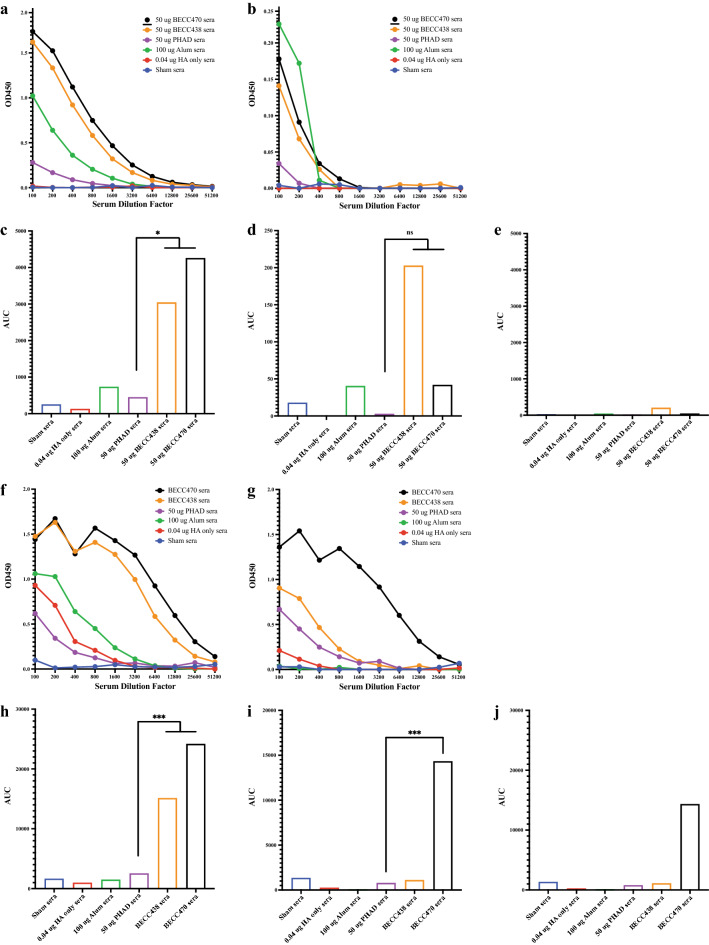
BECC adjuvants increase binding to conserved HA stalk in young but not aged mice. (**a**) ELISA (mean) total IgG binding to stalk-only HA. Pooled sera (by group) from day-28 prime-boost vaccinated young adult BALB/c mice vaccinated with 0.04 µg Cal/09 rHA protein in combination with 100 µg alum, 50 µg PHAD, 50 µg BECC438 or 50 µg BECC470 adjuvant. (**b**) ELISA (mean) total IgG binding to stalk-only HA. Pooled sera from day-28 prime-boost vaccinated aged (12-month-old) BALB/c mice vaccinated with 0.04 µg Cal/09 rHA protein in combination with 100 µg alum, 50 µg PHAD, 50 BECC438 or 50 µg BECC470 adjuvant. (**c**) AUC (mean) of pre-infection total IgG antibody titer curves of young adult mice in figure (**a**) above (BECC438 *p = 0.0208 and BECC470 *p = 0.0136). (**d**) AUC (Mean) of pre-infection total IgG antibody titer curves of aged mice in figure (**b**) above (ns). (**e**) AUC (Mean) of pre-infection total IgG antibody titer in aged mice in (**d**) scaled to young adult mice in (**c**). (**f**) ELISA (mean) total IgG binding to HA1-only HA from young adult BALB/c mice pre-infection day-28 serum. (**g**) ELISA (mean) total IgG binding to HA1-only HA from aged BALB/c mice pre-infection day-28 serum. (**h**) AUC (mean) of pre-infection total IgG antibody titer curves of young adult BALB/c mice in figure (**f**) above (BECC438 ***p = 0.0008 and BECC470 ***p = 0.0002). (**i**) AUC (mean) of pre-infection total IgG antibody titer curves of aged mice in figure (**g**) above (***p = 0.0005). (**j**) AUC (mean) of pre-infection total IgG antibody titer in aged mice in (**h**) scaled to adult mice in (**i**).

There is increasing interest in evaluating vaccines or adjuvants which are capable of increasing the breadth of the antibody response to influenza virus HA. Therefore, to assess adjuvant-induced production of HA stalk-reactive antibodies, we pooled (by vaccination group) day 28 sera collected from 6 to 8-week-old, female, BALB/c mice and used ELISA to measure total IgG binding to recombinant headless HA^21,23^. During vaccination, mice received prime (day 0) and boost (day 14) immunizations with 0.04 μg rHA alone or in combination with either 100 μg alum, 50 μg PHAD, 50 μg BECC438, or 50 μg BECC470 adjuvant. Total IgG antibody bound to HA stalk was low in sham and HA-only mice and slightly elevated in groups adjuvanted with alum and PHAD. However, there were significantly higher stalk-specific antibodies observed in groups adjuvanted with BECC438 (p = 0.0208) and BECC470 (p = 0.0136) as compared to PHAD (Fig. 4a,c).

As a comparator, we also tested stalk-reactive antibody production to the headless HA antigen in aged, 12-month-old, retired breeder BALB/c mice. Using the same prime-boost vaccination protocol as with young adult mice, pooled day 28 sera from aged mice was evaluated via ELISA to discern any age-altered antibody binding. There was very low IgG binding to recombinant stalk HA in aged mouse sera (no significant difference) (Fig. 4b,d) and when scaled to younger mice the total IgG bound to recombinant stalk protein was negligible (Fig. 4e).

Because there was BECC-adjuvanted vaccine protection in young adult mice^18^ and aged mice (Figs. 2g,h, 3g,h) we followed up stalk binding by testing antibody reactivity to commercially sourced HA1 antigen which includes the HA head domain, but lacks broadly cross-reactive stalk epitopes. In the sera of young adult mice, we not only found significantly higher levels of stalk antibody in BECC438 and BECC470-adjuvanted mice, but the same with HA1-only reactive antibody compared to PHAD (p = 0.0008 and p = 0.0002) (Fig. 4f,h). In aged mice, there was protection from weight loss and decreased pathology with very little stalk-reactive antibody present. In these aged mice, we hypothesize that sterilizing protection was provided by the robust HA1-only antibody which clearly shows a significant difference between the PHAD and BECC470 adjuvanted groups (p = 0.0005) (Fig. 4g,i), even when comparing production in aged mice to young adult mice (Fig. 4j).

## Discussion

With age, there is an increasing impairment of our natural immune response to infection^17,18^. This impairment brings with it a significant health care burden as our population continues to get older. Additionally, due to antigenic drift of seasonal influenza viruses and potential for future emergence of pandemic strains, flexible vaccine production for a robust and broadly protective response is greatly needed in both quantity and quality for all age groups^19^. The use of an immunostimulating adjuvant could be beneficial for the development of effective B and T cell responses with increased breadth and durability.

Several adjuvants have been used in humans to increase the immunogenicity of the accompanying antigen. The squalene-based oil-in-water emulsions, Addavax and MF59 have been used in approved vaccines as they have demonstrated increases in antibody titer when included. Each has an ability to modify the immune response after administration in a vaccine formulation. In this study, Aluminum salts (Alum) and synthetic Monophosphoryl Lipid A (called Phad) were used as adjuvants in our experiments. Addavax, a proprietary compound, and MF59 were not used as comparators due to their need for oil-in-water formulations. The BECC adjuvants allow for simple formulation and still high levels of immunogenicity when combined with antigen in a vaccine and are directly usable for pre-clinical experiments. Additional experiments using alternate BECC formulations will provide comparisons of immunogenicity for its paired antigen over other formulated adjuvants.

In our prime-boost vaccine study in aged mice, we showed robust protection from weight loss, superior antibody production, and decreased lung viral titers with the BECC-adjuvanted vaccine (Fig. 1). The BECC470 and other BECC adjuvants have previously been evaluated in vitro for their ability to stimulate a balanced immune response, leading to their selection as adjuvants for analysis^16^. We have found that using BECC470 as an adjuvant in an Influenza HA vaccine model leads to high protection levels in adult aged mice^18^. In aged mice, while a low dose of recombinant HA antigen (0.04 μg) in combination with BECC438 did not protect, this same low dose of BECC470-adjuvanted vaccine showed superior protection in mice compared to vaccine adjuvanted with alum, which is widely used in FDA-approved vaccine formulations for human use. BECC470-adjuvanted vaccine likewise outperformed the adjuvant PHAD, another TLR4 agonist. Results in aged mice show superior protection with BECC470 adjuvanted vaccine compared to alum and PHAD, but with surprising lack of protection with BECC438—previously shown to be protective in younger^18^. These data suggest that adjuvant selection may need to be tailored to specific risk-groups to maximize their ability to enhance protection. Mechanistic studies to precisely determine the mechanism of action, and profile of immune response elicited in different age groups will advance the future clinical use of novel adjuvant formulations.

Interestingly, with prime only vaccination using high-dose (5 μg) antigen adjuvanted with BECC438, we increased antibody production and recovered protection from weight loss in aged mice. In this same study, BECC470-adjuvanted vaccine mice maintained significantly higher H1-specific total and IgG specific antibody production and superior protection from weight loss compared to mice vaccinated with BECC438 (Fig. 2). The limited structural differences between BECC438 and BECC470 suggest different binding and signaling to MD2/TLR4 may be involved in this protection difference. Future studies looking at binding efficiency and signaling pathways would help shed light onto the specific causes of these differences and reasons for markedly higher antibody production and weight loss protection.

Vaccine effectiveness is often associated with antibody production (e.g., as measured in the hemagglutinin inhibition assay for influenza virus) but, this is not always associated with protection. We have shown that prophylactic passive transfer of antibodies in sera from mice vaccinated with BECC adjuvants provided only partial protection from weight loss (albeit at a significantly higher rate) as compared to all other vaccination groups (Fig. 3). Weight loss, however, is only one part of the protection. While viral lung titer was high in all groups and all passive transfer experiments, in addition to weight loss, there was a marked difference inflammation when antibody treatment was given post-infection. Furthermore, several studies have suggested that effective protection from influenza virus challenge requires cooperation between several immune effectors (i.e. T cells and antibodies)^21,22^. In a post-exposure therapeutic model, doubling the volume of antibody by administering 100 μL on days 1 and 3 after infection did improve inflammation in the BECC470 group, but did not in the BECC438 group. The bolus of antibody may have reduced virus titer at the cell surface post-infection while there was ongoing viral replication within cells. This is consistent with the method by which stalk-specific antibodies can confer protection in vivo: they recognize HA on the surface of infected cells, bind to stalk epitopes and recruit effector cells such as alveolar macrophages, which facilitate viral clearance and the resolution of infection^21,24^. Decreased protection with both BECC-adjuvanted serum passive transfers was likely due to the absence of an effective cellular response when compared to protection seen with vaccination. We have shown throughout our experiments that BECC adjuvant has a clear role in amplifying the quantity of antibody production. These passive transfer experiments indicate that while quantity is important for early protection, “fine tuning” of the B and T cell population along with other effector cells is likely important in robust protection. As reported by Goodwin et al*.*^25^, the T-cell response is especially important in the aged population due to a diminished antibody production.

In addition to stimulating neutralizing antibodies following infection or vaccination, antibodies with Fc-mediated effector function can also play an important role in conferring protection in vivo^24,26–28^. In recent years, it has been shown that non-neutralizing antibodies which target the HA stalk domain and are capable of mediating antibody-dependent cellular cytotoxicity (ADCC), antibody-dependent cellular phagocytosis (ADCP)and complement mediated cytotoxicity (CDC) are vitally important for breadth of protection. To shed light on BECC-adjuvanted vaccine’s role in immune stimulation, we evaluated the extent of antibody binding to recombinant headless HA protein. Stalk-reactive antibodies can result in broader and more robust protection from influenza virus challenge^28,29^. When we compared the sera from younger adult mice to aged mice, we found that BECC-adjuvanted sera in the younger cohort produced a robust stalk-reactive antibody response to the headless HA antigen. However, in the aged mice there was very little stalk-bound antibody in all groups (Fig. 4).

BECC adjuvanted vaccines afford improved binding of the HA stalk in young adult mice. Stalk reactivity can potentially lead to greater effector innate cell involvement and as a result, viral elimination. Protection by stalk antibodies in animal models can be achieved through several mechanisms: stalk Abs can be neutralizing, preventing viral fusion and/or egress, or non-neutralizing HA stalk antibodies can confer protection in vivo through engagement of Fc-mediated effector functions, such as ADCC or ADCP^24,27,30^. The reduced evolutionary rate for stalk makes it a good target for broad vaccines, potentially eliminating the need for annual influenza vaccination and stretching high quality immune protection over multiple seasons, or against emerging influenza viruses^4^. One caveat in these studies is that the mice used in these experiments were not previously seroconverted by either vaccination or infection to Influenza virus unlike humans. The presence of memory B cells and T cells recognizing Influenza virus antigen may alter the immune responses to both vaccination and the results of infection. In this naïve mouse model, we can piece apart the mechanistic differences that an adjuvant can have on protection, but we do not know how well BECC470 will enhance vaccination when an individual is already seroconverted.

While the goal of most Influenza vaccinations is to achieve sterilizing immunity through neutralizing antibody titers, there is a role for resident memory CD4 and CD8 T cell populations in rapidly controlling virus that has evaded neutralizing antibody. Adding to their value in the Influenza immune response, these T cell populations are known to provide heterosubtypic protection, responding to more conserved epitopes^31^. The use of adjuvants is known to shape the effector T cell response which directly influences the memory populations. Other adjuvants have been shown to more effectively generate CD4 and CD8 T cell resident memory in the context of vaccination^32^. As such, it will be important to evaluate how the cellular immune response is shaped following the use of BECC470 adjuvant, particularly in the aged population which is known to have measurably decreased cellular responses at baseline.

Future studies delineating the cytokine and immune cell response could shed light onto the BECC adjuvant effect and superior protection over alum and PHAD. While the BECC adjuvants have a clear role in amplifying the quantity of antibody production, passive transfer experiments indicate that other components of the immune response to immunization may contribute to increased protection. Future studies should focus on determining if BECC adjuvants stimulate cellular immune responses or increase breadth of reactivity against heterologous and even more divergent heterosubtypic influenza group 1 HAs such as avian H5. BECC adjuvants stimulate not only protective antibodies towards the head of HA but appear to drive broader protection and stalk-reactive antibodies that have the potential to engage APCs and FcγR’s for superior protective outcomes.

## Materials and methods

### Recombinant proteins and virus strains

rH1 (A/California/04/2009) with a C-terminal trimerization domain from fibritin foldon, used for immunizations, was expressed from baculovirus vectors in High Five cells (BTI-TN-5B1-4 cells; subclone from the Vienna Institute of Biotechnology^33,34^). For serological detection of H1-specific and HA stalk-specific antibody responses, recombinant H1 (A/California/04/2009) and MiniHA #4900 (based on H1 A/Brisbane/59/2007) were expressed in Expi293F cells, as previously described^21,23^. To avoid detection of antibodies binding the trimerization domain, both probes used for ELISA were engineered with an alternative C-terminal GCN4 isoleucine zipper trimerization domain from *Saccharomyces cerevisiae*. Recombinant HA1 domain (A/California/04/2009), was purchased from Sino Biologics does not feature a trimerization domain (#11055-V08H4). A wildtype pH1N1 influenza virus A/Netherlands/602/2009 (H1N1) (NL/09) was used for challenge experiments.

### Mice

All animal studies were approved by the University of Maryland School of Medicine Institutional Animal Care and Use Committee and were carried out at The University of Maryland School of Medicine Biohazard suite. Our study was carried out in compliance with the ARRIVE guidelines and all methods were carried out in accordance with all relevant guidelines and regulations.

### Adjuvanted vaccine protection in aged mouse model

To show protection from homologous challenge, aged (~ 12-month-old), female, retired breeder, BALB/c mice, purchased from Taconic Biosciences (Rensselaer, NY), were immunized intramuscular (IM) in the hind, caudal thigh with 100 mL vaccine solution at day 0 and day 14 (prime-boost schedule) with 0.04 µg of rHA or day 0 only (prime only schedule) with 5 µg of rHA of influenza A/California/04/2009 (pandemic H1N1) (Cal/09) adjuvanted with either 100 µg of alum, 50 µg PHAD, 50 µg BECC438, or 50 µg BECC470 (HA antigen and BECC adjuvants previously reported in^16^). On day 28 all groups were inoculated i.n. with 32,000 PFU of homologous A/Netherlands/602/2009 (H1N1) (NL/09) virus and weighed daily for seven days. Mice that fell below 70% of starting weight were euthanized per IACUC protocol. On day 7 post-infection mice were harvested for blood and lung tissue following IACUC protocols.

### Serum transfer protection from homologous challenge

As reported in Gregg et al.^13^, to assess the role of IgG in the protection induced by the vaccines with and without adjuvant, wild-type, 6–8 week-old, female, BALB/c mice (Charles River) were immunized IM in the hind, caudal thigh at day 0 and day 14 with 0.04 µg of rHA from influenza A/California/04/2009 (pandemic H1N1) (Cal/09) plus either 100 µg of alum, 50 µg PHAD, 50 µg BECC438, or 50 µg BECC470 or with unadjuvanted antigen or phosphate buffered saline (PBS). At day 28, mice were anesthetized using an isoflurane/oxygen mixture and bled via cardiac puncture. The serum within each group was pooled and frozen at − 80 °C. First, to assess antibody protection pre-infection, 100 µL of untreated sera was injected IP into each non-anesthetized, naïve mouse (by group). The mice were allowed to rest for two hours and then they were inoculated i.n. with 3200 PFU of homologous A/Netherlands/602/2009 (H1N1) (NL/09) virus and weighed daily for seven days. Mice that fell below 70% of starting weight were euthanized per IACUC protocol. On day 7 post-infection mice were harvested for blood and lung tissue following IACUC protocols. Next, to assess antibody protection from homologous challenge post-infection, we inoculated groups of mice i.n. with 3200 PFU of IAV NL/09 virus on day 0, then on day 1 and day 3 each post-infected, we IP-injected 100 µL serum from each above vaccination group (including non-immunized control groups) and weighed daily for seven days following IACUC protocols. On day 7 post-infection mice were harvested for blood and lung tissue following IACUC protocols.

### HA specific antibody titers by ELISA

Briefly (as described in detail in^16^), total IgG, IgG2a and IgG1 antibody titers were determined by ELISA in sera collected on day 28 of the vaccination schedule (pre-infection). Depending on the experiment, 50 μL of either full trimer Cal/09 rHA or group 1 H1 stalk Mini-HA or HA1 Subunit (Sino Biologicals #11055-V08H4) protein-coated 96-well plates (Nunc Maxisorp #44240421) were incubated at 4 °C overnight, washed, and then blocked in goat serum/milk/PBS-T for 1 h^21,23^. Blocking buffer was then removed and duplicates of 100 μL of serum, diluted in blocking buffer, was added in a ten point, two-fold dilution series starting with a 1:100 dilution. After two hours of incubation at RT, plates were washed and secondary antibody for either total IgG (goat anti-mouse IgG, KPL-474-1802, Kirkegaard and Perry Laboratories, Gaithersburg, MD), IgG1 (1070-05, Southern Biotech, Birmingham, AL) or IgG2a (1080-05, Southern Biotech, Birmingham, AL) was added for one hour at RT. Plates were washed again and 50 μL of room temperature BD OptEIA 3,3′,5,5′-tetramethylbenzidine (TMB) substrate (BD Biosciences, San Jose, CA) was added to the plates, incubated for 10 min at RT and the reaction stopped by adding 50 μL of 3 M HCl. An OD450 value was determined using a BioTek Instruments Synergy HTX plate reader using BioTek Instruments Gen5 software (BioTek Instruments, Winooski, VT). All analyses and graphs were made using Prism 9 for Mac OS X (GraphPad Software, Inc., La Jolla, CA). Antibody titer (mean + SEM) and area under the curve (AUC) (mean ± standard error, SEM) are individual mice readings with the blank subtracted from the readout. Prism computes the area under the curve using the Gagnon trapezoid rule with mean and SEM reported as a standard error and confidence interval for the AUC.

### Lung tissue histology

Lung tissue fixed in 4% paraformaldehyde (Sigma-Aldrich, St Louis, MO) was processed for hematoxylin and eosin (H&E) slides (University of Maryland—Baltimore EM and Histology Laboratory) to assess the histopathology and potential resolution of viral infection. Representative stained tissue sections were selected to most accurately portray the condition of the lungs within each group of vaccinated mice (Supplemental Figs. 1–4). A single pathologist assessed the selected sections for the presence of alveolar consolidations, peribronchial and perivascular inflammatory infiltrate, and bronchial necrosis then scored them for overall inflammation using the following scoring parameters: 0 = no inflammation, 1 = scant inflammation (< 5% multifocal or one small focus); 2 = prominent inflammation, < 25% parenchyma; 3 = pronounced inflammation, 25–50% parenchyma; 4 = diffuse inflammation, 50–75% parenchyma; 5 = widely diffuse inflammation, > 75% parenchyma. The scores of each group of vaccinated mice were averaged for comparison. All analyses and graphs (mean + SEM) were made using Prism 9 for Mac OS X (GraphPad Software, Inc., La Jolla, CA).

### Plaque assays for lung virus titer

Plaque assays were used to determine the lung IAV titers from mice euthanized on day 7 post infection. Madin-Darby canine kidney (MDCK) cells were grown to confluence overnight at 37 °C on 6-well plates and a dilution series from 1 × 10^–1^ to 1 × 10^–6^ of lung homogenate were added to the wells and allowed to infect the cells for 1 h with period rocking. After one hour, cells were washed with Dulbecco's Modified Eagle Medium (DMEM) (Quality Biological, Gaithersburg, MD) supplemented with 1 × penicillin/streptomycin (Gemini Bio- Products, Sacramento, CA) and 1% l-glutamine (Gibco/Life Technologies, Grand Island, NY). A 3-mL overlay of SeaKem LE Agarose (Lonza, Rockland, ME) mixed with 1:500 dilution of Tosyl phenylalanyl chloromethyl ketone (TPCK)-treated trypsin (Sigma-Aldrich, St Louis, MO) in 2 × MEM (Quality Biological, Gaithersburg, MD) was used to cover the cells in each well and the plates were incubated at 37 °C for three days. After this incubation period the agarose was removed, cells were fixed with 70% ethanol then stained with 0.05% crystal violet. Plaques were visualized and counted manually. All analyses and graphs (mean + SEM) were made using Prism 9 for Mac OS X (GraphPad Software, Inc., La Jolla, CA).

## Supplementary Information

Supplementary Legends.

Supplementary Figure 1.

Supplementary Figure 2.

Supplementary Figure 3.

Supplementary Figure 4.

## Data Availability

The datasets used and/or analyzed during the current study are available from the corresponding author on reasonable request.
