# Supplementary material for: Enhancing the protection of influenza virus vaccines with BECC TLR4 adjuvant in aged mice
Source: Sci Rep. 2023 Jan 13;13:715. doi: 10.1038/s41598-023-27965-x (PMC9838488; doi:10.1038/s41598-023-27965-x)
Supplement: Supplementary file 1 — Supplementary Legends. [file 41598_2023_27965_MOESM1_ESM.docx]

**Supplementary Figure legends**

**Supplemental Figure 1.** Mice prime-boost vaccinated with BECC470 adjuvant have decreased lung inflammatory response against influenza A infection. H&E-stained lung sections of 12-month-old BALB/c mice 7 days post infection shown. Vaccination groups (with 32,000 PFU IAV NL/09 infection as indicated) shown in panels from left to right and top to bottom include: PBS only, 0.04 μg HA only, 0.04 μg HA/100 μg Alum, 0.04 μg HA/50 μg PHAD, 0.04 μg HA/50 μg BECC438 and 0.04 μg HA/50 μg BECC470. 10X magnification.

**Supplemental Figure 2.** BECC470 adjuvanted prime only vaccination reduces lung inflammation. H&E-stained lung sections of 12-month-old BALB/c mice 7 days post infection shown. Vaccination groups (with 32,000 PFU IAV NL/09 infection as indicated) shown in panels from left to right and top to bottom include: PBS only, 5 μg HA only, 5 μg HA/50 μg BECC438 and 5 μg HA/50 μg BECC470. 10X magnification.

**Supplemental Figure 3.** Pre-infection passive transfer of 100 μL BECC adjuvanted sera reduces lung inflammation. H&E-stained lung sections of 6–8-week-old BALB/c mice 7 days post infection shown. Vaccination group sera (with 3200 PFU IAV NL/09 infection as indicated) shown in panels from left to right and top to bottom include: No sera with no infection, no sera with infection, sham sera with infection, 0.04 μg HA only sera with infection, 0.04 μg HA/Alum sera with infection, 0.04 μg HA/PHAD sera with infection, 0.04 μg HA/50 μg BECC438 sera with infection and 0.04 μg HA/50 μg BECC470 sera with infection. 10X magnification.

**Supplemental Figure 4.** Post-infection passive transfer of 200 μL BECC470 adjuvanted sera further reduces lung inflammation. H&E-stained lung sections of 6–8-week-old BALB/c mice 7 days post infection shown. Vaccination group sera (with 3200 PFU IAV NL/09 infection as indicated) shown in panels from left to right and top to bottom include: No sera with no infection, no sera with infection, 0.04 μg HA/50 μg BECC438 sera with infection and 0.04 μg HA/50 μg BECC470 sera with infection. 10X magnification.
