# Supplementary figures and images for: Enhancing the protection of influenza virus vaccines with BECC TLR4 adjuvant in aged mice
Source: Sci Rep. 2023 Jan 13;13:715. doi: 10.1038/s41598-023-27965-x (PMC9838488; doi:10.1038/s41598-023-27965-x)

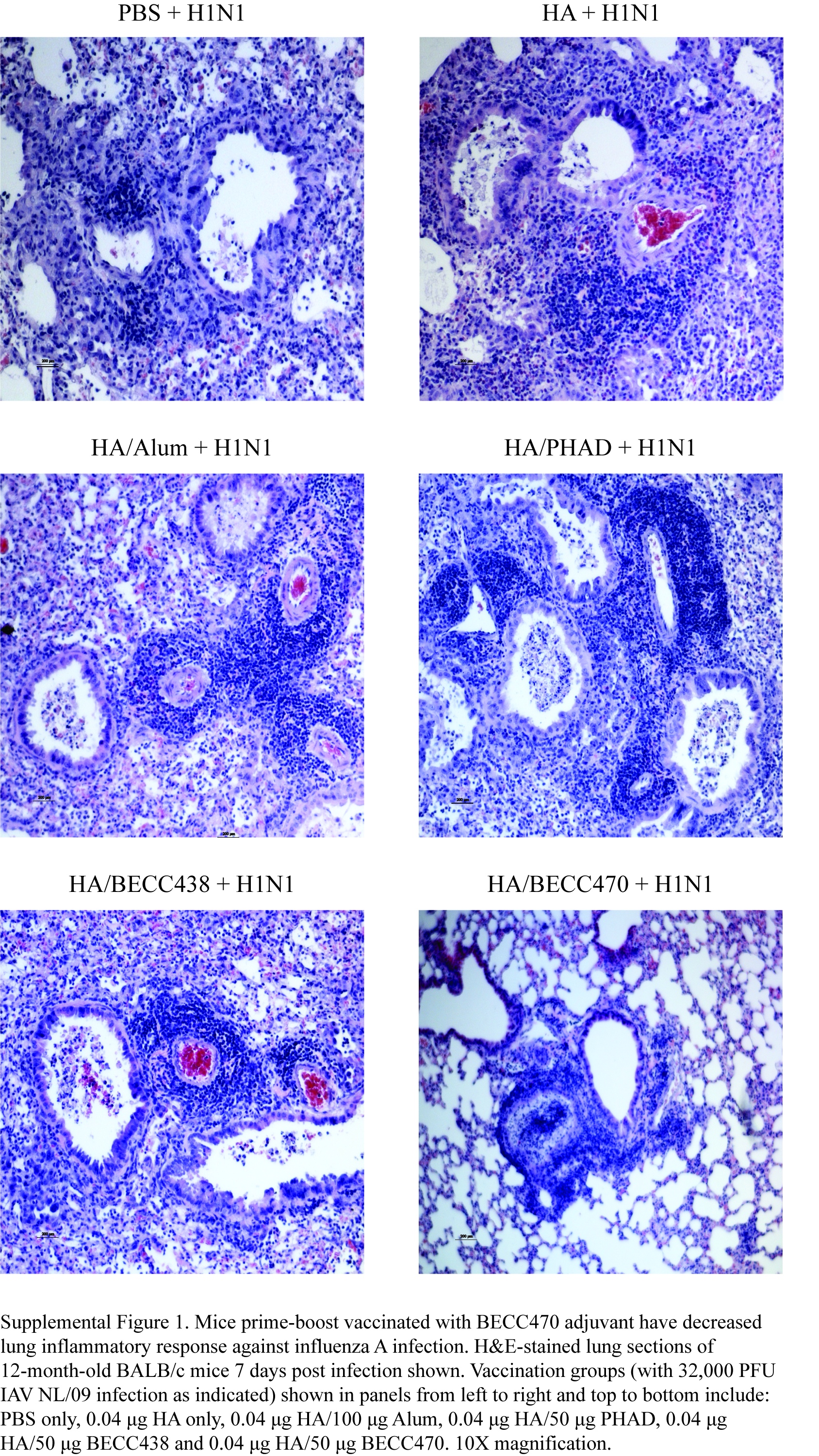

Supplement: Supplementary file 2 — Supplementary Figure 1. [file 41598_2023_27965_MOESM2_ESM.tif]

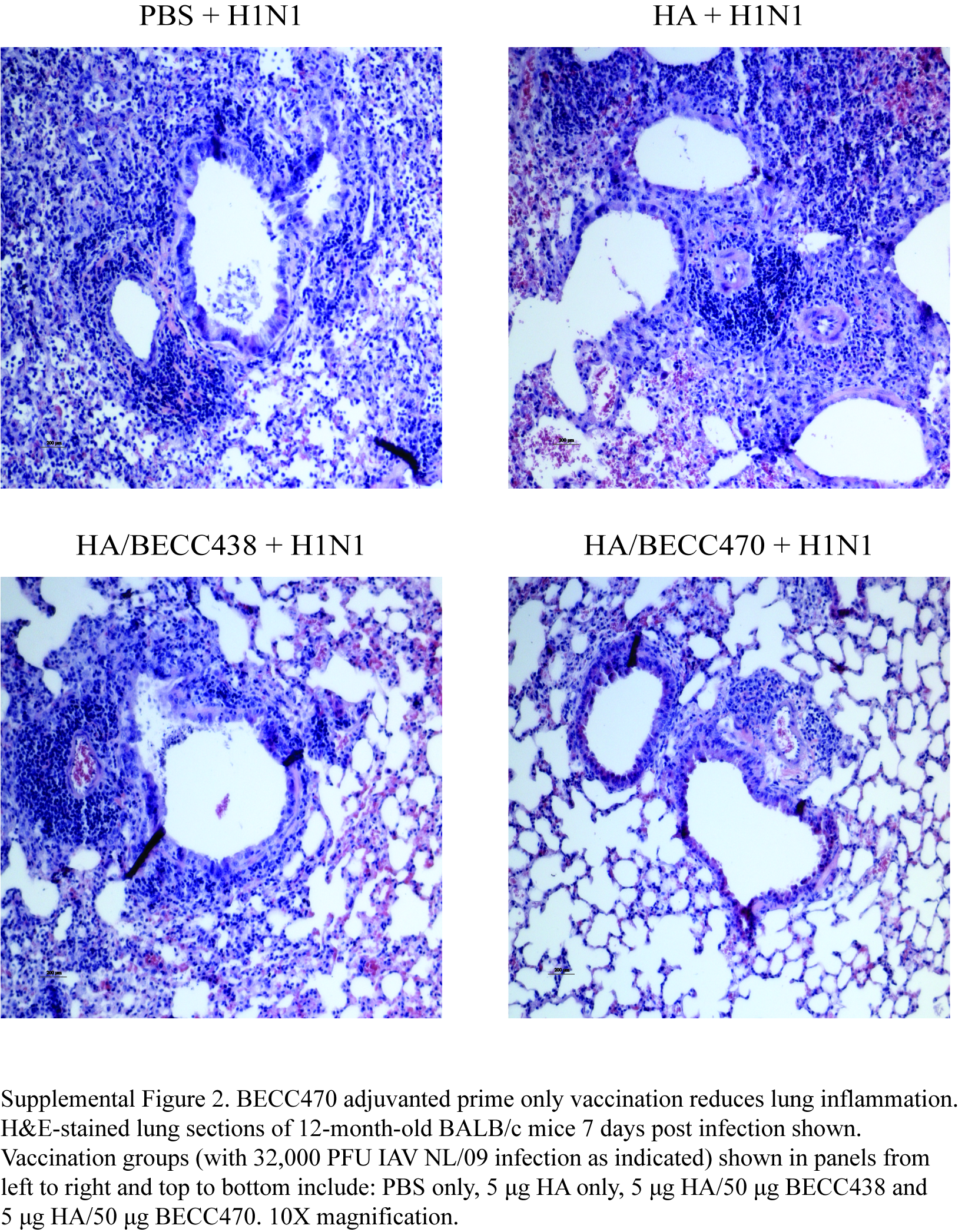

Supplement: Supplementary file 3 — Supplementary Figure 2. [file 41598_2023_27965_MOESM3_ESM.tif]

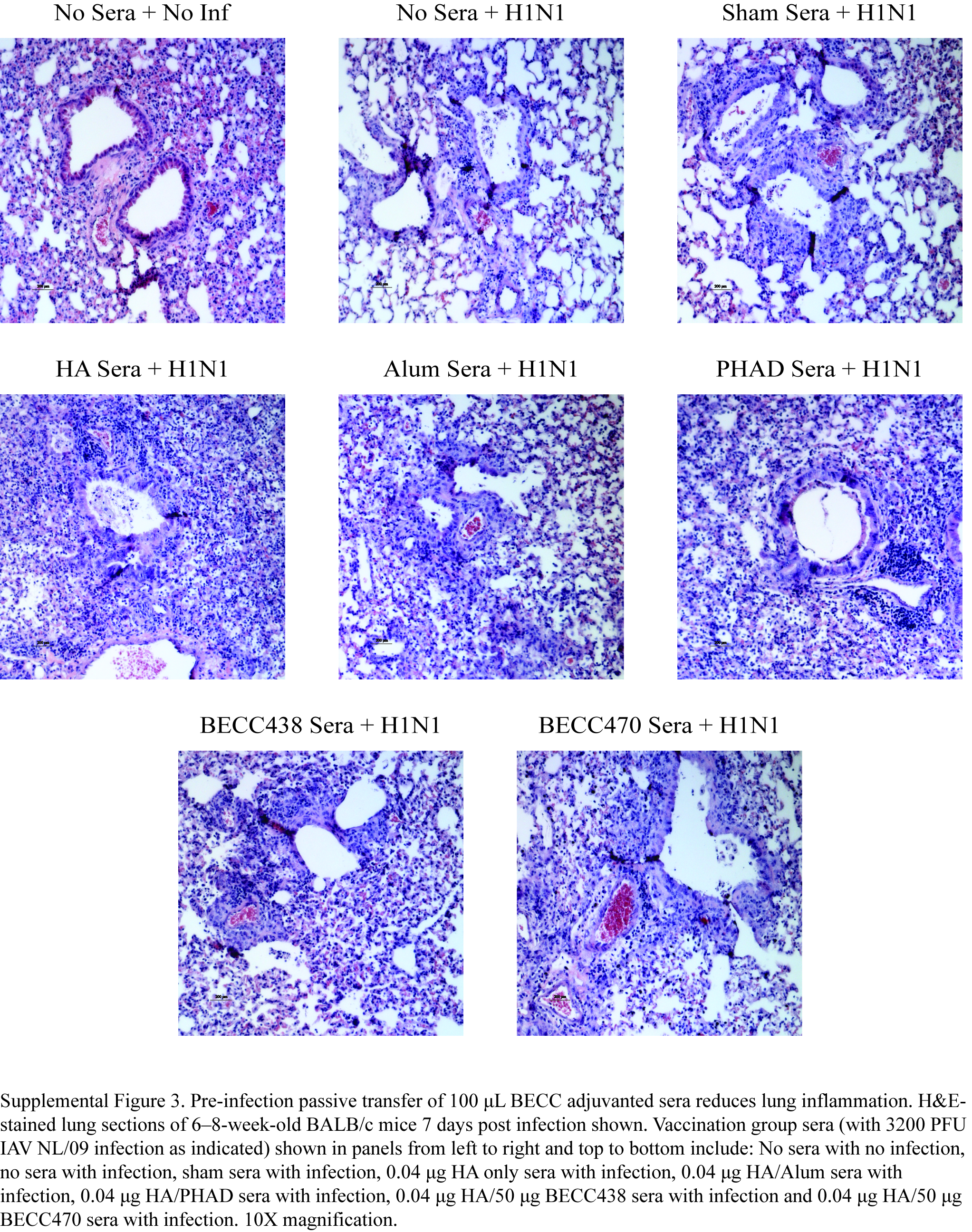

Supplement: Supplementary file 4 — Supplementary Figure 3. [file 41598_2023_27965_MOESM4_ESM.tif]

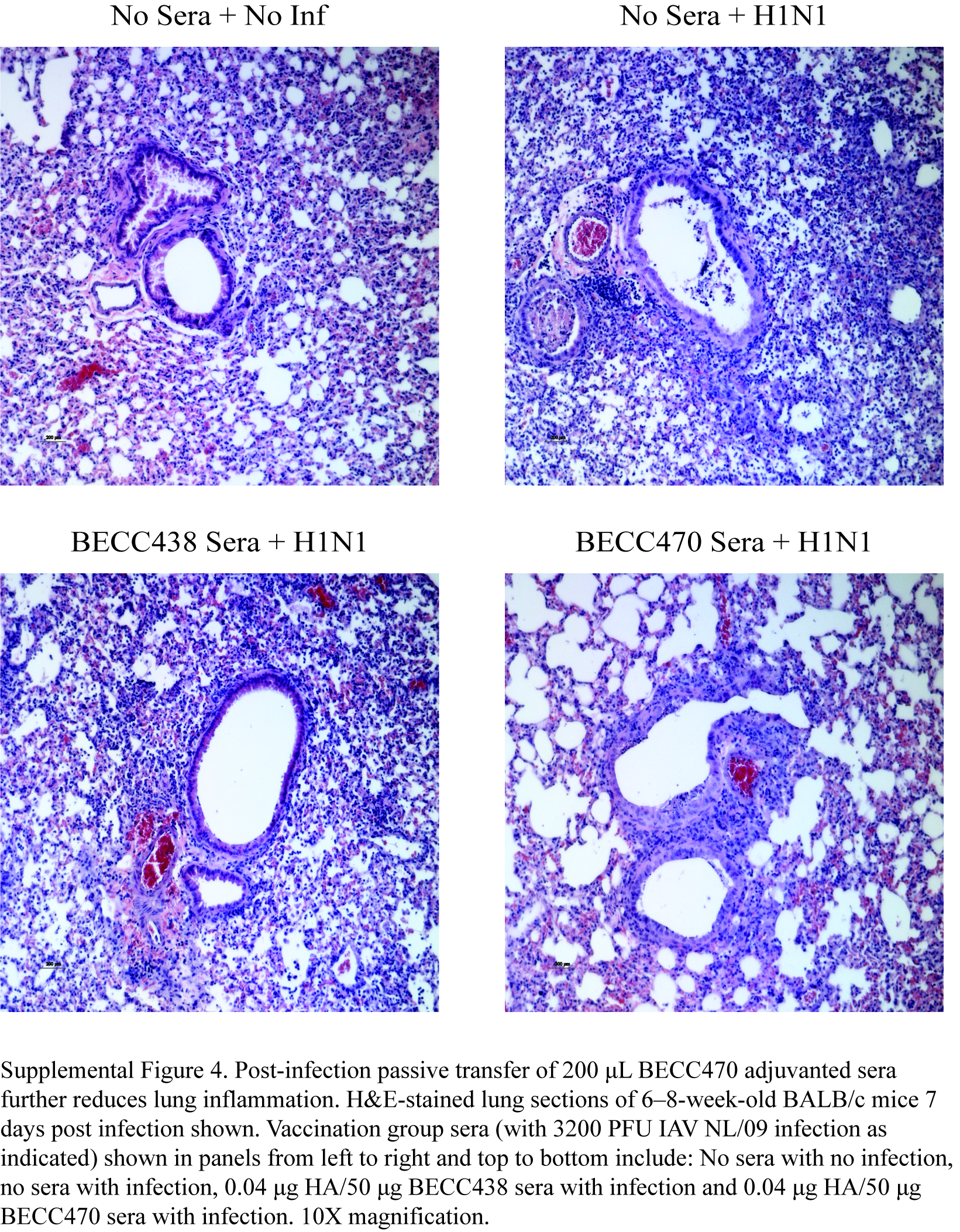

Supplement: Supplementary file 5 — Supplementary Figure 4. [file 41598_2023_27965_MOESM5_ESM.tif]
